# Characterization of the Soluble NSF Attachment Protein gene family identifies two members involved in additive resistance to a plant pathogen

**DOI:** 10.1038/srep45226

**Published:** 2017-03-24

**Authors:** Naoufal Lakhssassi, Shiming Liu, Sadia Bekal, Zhou Zhou, Vincent Colantonio, Kris Lambert, Abdelali Barakat, Khalid Meksem

**Affiliations:** 1 Department of Plant, Soil and Agricultural Systems, Southern Illinois University, Carbondale, IL 62901, USA; 2 Department of Crop Sciences, University of Illinois, Urbana, IL 61801, USA; 3 Department of Biology, University of South Dakota, Vermillion, SD 57069, USA

## Abstract

Proteins with Tetratricopeptide-repeat (TPR) domains are encoded by large gene families and distributed in all plant lineages. In this study, the Soluble NSF-Attachment Protein (SNAP) subfamily of TPR containing proteins is characterized. In soybean, five members constitute the *SNAP* gene family: *GmSNAP18, GmSNAP11, GmSNAP14, GmSNAP02,* and *GmSNAP09*. Recently, *GmSNAP18* has been reported to mediate resistance to soybean cyst nematode (SCN). Using a population of recombinant inbred lines from resistant and susceptible parents, the divergence of the *SNAP* gene family is analysed over time. Phylogenetic analysis of *SNAP* genes from 22 diverse plant species showed that *SNAPs* were distributed in six monophyletic clades corresponding to the major plant lineages. Conservation of the four TPR motifs in all species, including ancestral lineages, supports the hypothesis that *SNAPs* were duplicated and derived from a common ancestor and unique gene still present in chlorophytic algae. Syntenic analysis of regions harbouring *GmSNAP* genes in soybean reveals that this family expanded from segmental and tandem duplications following a tetraploidization event. qRT-PCR analysis of *GmSNAPs* indicates a co-regulation following SCN infection. Finally, genetic analysis demonstrates that *GmSNAP11* contributes to an additive resistance to SCN. Thus, *GmSNAP11* is identified as a novel minor gene conferring resistance to SCN.

The majority of crop species appears to be polyploids as a result of duplication or hybridization events. It is generally accepted that polyploidy has conferred distinct advantages to the development of agronomically important traits^1^,^2^,^3^. Polyploidization, for example, has been associated with an increased size of harvested organs, novel gene interactions leading to new traits, and the formation of new crop species^1^. In the plant model *Arabidopsis thaliana*, at least four different large-scale duplication events occurred 100 to 200 million years ago, favouring the diversification of this species^4^. Recently, numerous studies have reported large segmental duplication events and subsequent divergent selection across many gene families in soybean^5^,^6^,^7^. Soybean has a paleopolyploid genome and nearly 75% of predicted soybean genes are present in multiple copies due to two duplication events that occurred 13 and 59 mya^8^.

In recent years, proteins containing TPRs have been shown to be essential for responses to hormones such as ethylene, cytokinin, gibberellin, salicylate, and auxin in *Arabidopsis*. Thus, proteins containing TPRs are emerging as essential determinants for signal transduction pathways^9^. Several studies have reported that proteins containing TPRs are involved in a plethora of cellular functions including cell cycle regulation, neurogenesis, and mitochondrial/peroxisomal protein transport^10^,^11^. Interestingly, mutations in TPR proteins have been found to produce several human diseases, indicating essential roles in cell function. Importantly, the TPR domain facilitates specific interactions with a partner protein(s)^11^. Moreover, TPR domains also play important roles in aspects of plant development; being essential for gametophytic viability as well as root growth and integrity under osmotic stress^12^. In addition, a different member of TPR containing proteins, *TTL3*, was found to interact with the constitutively active VH1/BRL2, a protein homologous to the brassinosteroid receptor BRI1, and play a role in vasculature development^13^. There are a large number of proteins containing the TPR motif, and they are found in many organisms, including humans, yeast, bacteria, and plants^10^. The TPR gene family is divided into several subfamilies including tetratricopeptide thioredoxin like (TTL), Cyclophilin (CYP), and Soluble NSF attachement proteins (SNAP).

SNAPs have been widely studied in both plants and animals. SNAP protein, a member of soluble NSF attachment protein receptor (SNARE) complex, has been reported to be involved in vesicular trafficking, plasma membrane stability, cytokinesis (involving KNOLLE), calcium binding (involving Synaptotagmin), membrane repair, and human genetic diseases including certain cancers^14^,^15^,^16^,^17^,^18^. Additionally, an α-SNAP has also been linked to disease resistance in plants^19^,^20^. SNAPs are characterized by the presence of a tetratricopeptide repeat (TPR) domain. The TPR domain was identified and named in 1990, with a name denoting the 34 amino acids comprising the basic repeat and was reported to be involved in the cell cycle in yeast^21^,^22^. Proteins do not normally contain an individual TPR motif, but consist of three to 16 tandem repeats that can be grouped or dispersed throughout the protein^10^,^23^. In soybean, the *SNAP* gene family is composed of five members; of which *GmSNAP18* is required for SCN resistance^20^. However, the other *GmSNAP* members were not investigated for their role in SCN resistance.

Soybean (*Glycine max* (L) Merr.) is considered one of the most economically important crops worldwide. It is a valuable source of protein, edible oil, and biodiesel, and represents more than 56% of the world’s oilseed production (http://SoyStats.com, 2016). Soybean production is severely endangered by diseases such as soybean cyst nematode (*Heterodera glycines* Ichinohe), a microscopic worm which causes over $1.2 billion yield losses annually in the U.S alone^24^. Planting resistant cultivars is the preferred disease management strategy against SCN. Two types of SCN resistant soybean lines have been used by soybean breeders, the PI88788 type of resistance which requires three genes together at the *rhg1* locus for its function: a Soluble NSF Attachment Protein (α-SNAP), an Amino Acid Transporter (AAT) and a Wound-Inducible domain (WI12)^20^; and the Peking type of resistance requires two genes: the *GmSNAP18* at the *rhg*1 locus and the *GmSHMT08* at the *Rhg*4 locus^25^,^26^. However, the molecular mechanisms of how SNAP proteins mediate SCN resistance remain unclear. Recently, it has been described that elevated expression of resistance-type *Rhg1 α-SNAP* negatively affected the abundance of SNARE-recycling 20 S complexes, disrupting vesicle trafficking, and induced elevated abundance of NSF causing cytotoxicity^27^. However, expression of other loci encoding a canonical wild-type α-SNAPs counteracted the cytotoxicity of resistance-type Rhg1 α-SNAP^27^. Furthermore, a SCN gene encoding a bacterial-like protein containing a putative SNARE domain (HgSLP-1), an esophageal-gland protein that is secreted by the nematode during plant parasitism, has been suggested to physically interact with the Rhg1 α-SNAP in SCN resistance^28^. The authors suggested that HgSLP-1 protein may function as an avirulence protein and it helps SCN evade host defenses when absent.

In this study, we conducted a detailed phylogenetic and structural characterization of the *GmSNAP* subfamily from various species. Furthermore, we tested the contribution of soybean segmental duplications of *SNAP* and investigated if they originated from an ancestral gene in plants with subsequent duplication events. Results obtained suggest that this family evolved from an early land plant ancestor, and was subject to duplications followed by subfunctionalization and neofunctionalization events. Expression profiling and functional analysis of *GmSNAP* genes were also performed. Finally, we demonstrate that in addition to *GmSNAP18*, the *GmSNAP11* is a novel minor gene in SCN resistance, contributing to an additive effect.

## Results

### Duplication of *GmSNAP* genes in soybean genome

**Figure 1:**
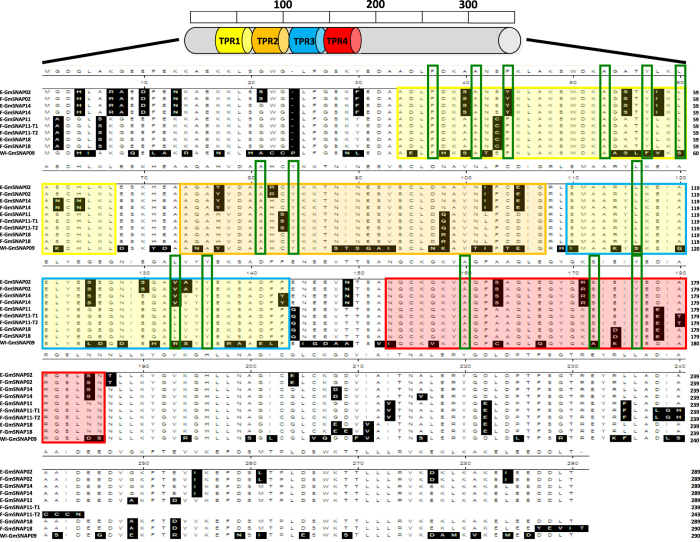
Comparative analysis of GmSNAP’s TPR proteins. Amino acid alignment for the four TPR domains of the five predicted GmSNAP protein members in soybean. *In silico* analysis showing a high similarity between TPR domains. Yellow, orange, blue, and red boxes present the details of the alignment of the TPR1, TPR2, TPR3, and TPR4 domains, respectively. Green boxes indicate carboxylate clamp residues highly conserved in TPR domains. The alignment shows a high similarity between GmSNAP18 and GmSNAP11, followed by GmSNAP14 and GmSNAP02. However, GmSNAP09 presented the highest polymorphisms.

*In silico* analysis reveals that SNAPs constitute a family of proteins with a common modular architecture containing four tetratricopeptide repeat (TPR) motifs conserved and distributed in specific positions throughout the sequence (Fig. 1). The TPR motifs are common modules in molecular chaperones and are required for the establishment of protein–protein interactions during the formation of multi-protein complexes^11^. Extensive searches employing a variety of sequenced genomes using the typical distribution of the four TPR motifs of the soybean SNAPs failed to identify any members of this protein family in red algae (*Hemiselmis andersenii*). However, SNAPs were found to be present in all plant genomes analyzed, including chlorophytic algae (Supplementary Table S2).

Investigation of the Williams 82 soybean genome indicates that the *GmSNAP* gene family is composed of five members located on chromosomes 02 (Glyma.02g260400), 14 (Glyma.14g054900), 11 (Glyma.11g234500), and 18 (Glyma.18g022500), all encoding 289 amino acid (aa) proteins, except one in chromosome 09 (Glyma.09g279400) encoding a 290aa protein. *GmSNAP18* was previously reported to be involved in SCN resistance, along with an amino acid transporter (*AAT*) and a wound inducible protein (*WI12*)^20^. However, no study has reported any function of the other four *GmSNAP* members (*GmSNAP11, GmSNAP14, GmSNAP02, GmSNAP09*) in SCN resistance.

In order to test the contribution of the soybean duplication events in the number of *SNAP* genes, the soybean genome was analysed for duplicated chromosomal segments containing *GmSNAP*s using the Plant Genome Duplication Database^29^,^30^,^31^. Using the locus surrounding *GmSNAP18* as bait, three independent duplicate blocks (±100 kb) were discovered to harbour *SNAP11, SNAP14*, and *SNAP02* genes (Table 1, Supplementary Fig. S1). *GmSNAP*s intragenome syntenic relationship calculations for all the conserved genes surrounding *GmSNAPs* reveal that the *GmSNAP* duplication between chr18 and chr11 belongs to a very large inverted duplicated segments containing 386 additional conserved duplicated genes or anchors (Supplementary Table S2). However, *GmSNAP* duplication between chr18/chr14, and chr18/chr02 was not as conserved, with only 72 and 23 duplicated genes or anchors retained, respectively (Supplementary Table S2). Syntenic analysis showed that *GmSNAP18* and *GmSNAP09* were not located on duplicated blocks together. Similarity analyses showed that the GmSNAP09 protein shared the lowest identity with GmSNAP18 (only 68%), as compared to the other members GmSNAP02, GmSNAP14, and GmSNAP11, which shared 84.8%, 86.9%, and 92.4% identity with GmSNAP18, respectively (Fig. 1).

Because *GmSNAP18/GmSNAP11* and *GmSNAP14/GmSNAP02* blocks shared the highest amount of conserved genes (with 386 and 37 anchors) (Supplementary Table S2), this finding suggests that *GmSNAP18/GmSNAP11* and *GmSNAP14/GmSNAP02* were the result of a recent duplication event (13 mya)^8^. In addition, *Glycine max* intraspecific synteny data in Soybase suggests that *GmSNAP* on chromosome 18 was present in the ancient duplication event; however, *GmSNAP* on chromosome 11 has appeared only in the recent duplication.

### *In silico* and structural analysis of GmSNAP proteins reveals that *GmSNAP18* and *GmSNAP11* have conserved the carboxylate clamp residues

Within a TPR motif, eight amino acids at positions 4 (W/L/F), 7 (L/I/M), 8 (G/A/S), 11 (Y/L/F), 20 (A/S/E), 24 (F/Y/L), 27 (A/S/L), and 32 (P/K/E) are conserved and important in maintaining the α-helical structures^32^. *In silico* analysis showed that TPR1 of both GmSNAP18 and GmSNAP11 presents six conserved amino acids, 4 (F), 8 (A), 11 (F), 20 (A), 24 (Y), and 27 (L); TPR2 presents two amino acids conserved at positions 8 (A) and 11 (Y); TPR3 presents three conserved amino acids at positions 7 (L), 24 (L), and 27 (Y); and TPR4 presents three conserved amino acids at positions 8 (A), 20 (S), and 24 (Y) (Fig. 1). This structural analysis supports the functionality of the TPR domain in recognizing its target proteins^32^. However, GmSNAP02 and GmSNAP14 did not conserve the F11Y and L24V in the TPR1 and TPR3, respectively (Fig. 1). Interestingly, GmSNAP09 did not conserve these carboxylate clamp residues in most TPR domains. Positions 24 (F24Y) and 27 (L27S) in TPR1, positions 7 (L7S) and 24 (L24R) in TPR3, and 20 (S20A) in TPR4 were not conserved. In addition, GmSNAP09 presented the highest number of polymorphisms compared to GmSNAP18 and GmSNAP11 in TPR1 (35%), TPR2 (44%), TPR3 (41%), and TPR4 (26%) (Supplementary Table S3), followed by GmSNAP14 (8 to 20%) and GmSNAP02 (11 to 17%). No polymorphisms were observed between GmSNAP18 and GmSNAP11 at TPR1 and TPR3, only one polymorphism at TPR2 (2%) and two polymorphisms at TPR4 (5%) were present. However, no polymorphisms affected the conserved carboxylate clamp residues. Thus, GmSNAP11 share the most identity to GmSNAP18.

### Evolution of *GmSNAP* gene family members

**Figure 2:**
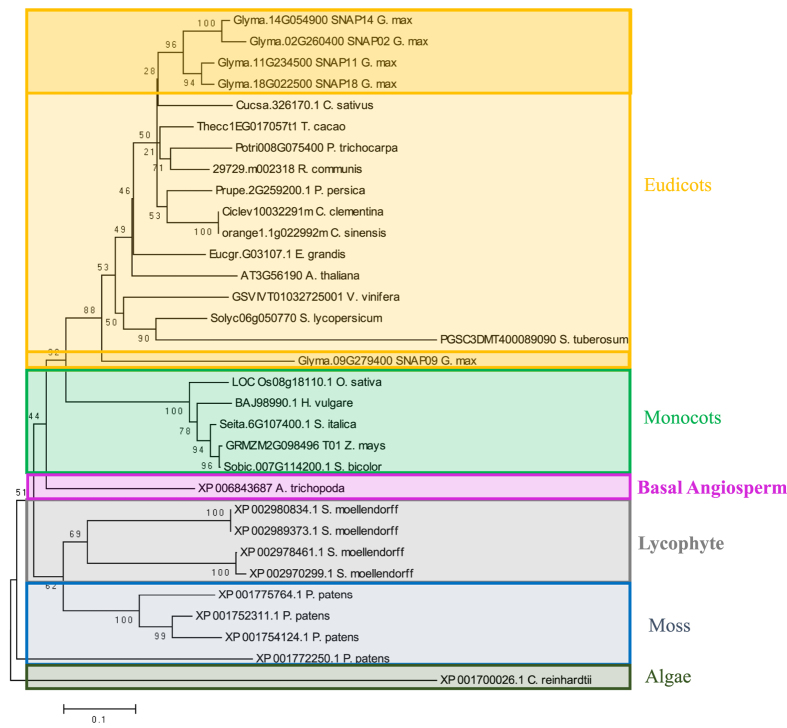
Phylogenetic tree of SNAP from 22 plants species. All SNAP proteins identified in six model plants; *C. reinhardtii* (algae), *P. patens* (moss), *S. moellendorfii* (lycophyte), *Amborella* (basal angiosperm), *O. sativa* (monocot), and *A. thaliana* (eudicot), in addition to *G. max* (soybean), were included in the phylogenetic analysis. However, only the SNAP proteins most similar to soybean GmSNAP18, were included from the rest of the other species. The phylogenetic tree was generated using MEGA4 software package and the ClustalW algorithm, and calculated using the neighbor-joining method. The tree bootstrap values are indicated at the nodes (n = 1000).

Phylogenetic analysis was conducted to elucidate the evolution of the *GmSNAP* gene family in soybean. The analysis showed that SNAPs were distributed in six subclades corresponding to eudicots, monocots, basal angiosperms, lycophytes, moss, and chlorophytic algae. This suggests that GmSNAPs derived from a common ancestor as *SNAP* genes are distributed in all plant lineages (Fig. 2). Monocot, eudicot, and basal angiosperm SNAPs were separately grouped from ancestral SNAPs from *S. moellendorfii, P. patens*, and *C. reinhardtii*. We will consider the *C. reinhardtii* SNAP as the root of this phylogenetic tree.

From the phylogenetic analysis, four GmSNAPs clustered together with GmSNAP18/GmSNAP11 and GmSNAP14/GmSNAP02 localized in subclades. Interestingly, GmSNAP09 did not cluster with the other GmSNAPs. This suggests that this gene diverged from the others or has become pseudogenized. Branch length distances suggest that GmSNAP18 is the most closely related to the ancestral SNAPs, and GmSNAP14 is more closely related to the ancestral SNAPs than GmSNAP02. Furthermore, the large conserved anchor number of GmSNAP18 from the syntenic analysis suggests that GmSNAP18 may be most similar to the ancestral SNAP, which also gave rise to GmSNAP14 in the oldest duplication event (59 mya), followed by the divergence of GmSNAP11 from GmSNAP18 and GmSNAP02 from GmSNAP14 in the most recent duplication event (13 mya) (Supplementary Table S2).

**Figure 3:**
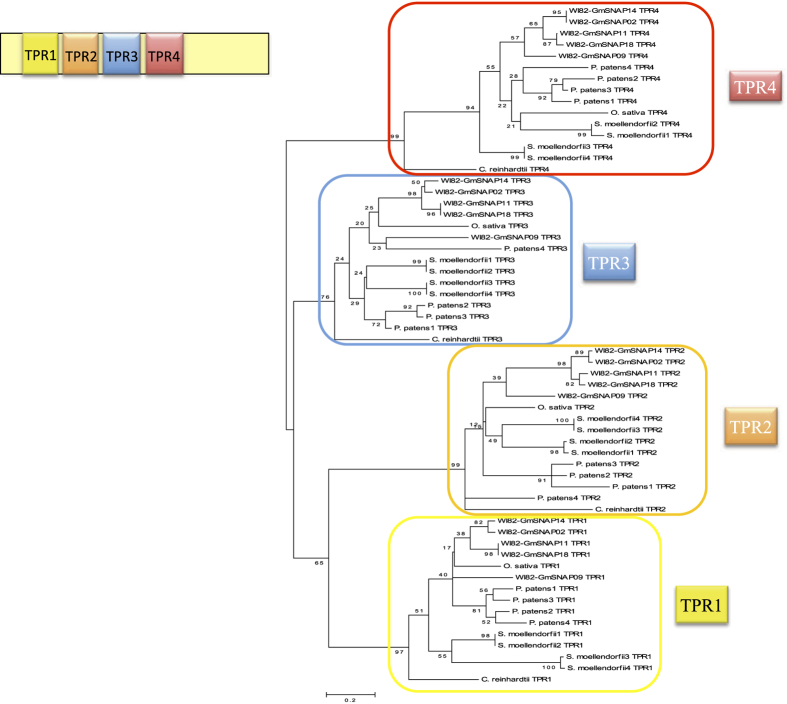
Phylogenetic relationships among individual TPR motifs in GmSNAP. All GmSNAPs in soybean contain 4 TPR motifs (TPR1, TPR2, TPR3, and TPR4). The unrooted tree was generated using the four TPR motifs sequences from the 5 soybean SNAP proteins, the SNAP orthologs from monocots and eudicots, and from phylogenetically distant species such algae, moss, and lycophyte. The phylogenetic tree was generated using MEGA4 software package and the ClustalW algorithm, and calculated using the neighbor-joining method. The tree bootstrap values are indicated at the nodes (n = 1000).

Next, we investigated whether *SNAP* genes originated from an ancestral gene in plants with subsequent duplications or resulted from convergent evolution. Because TPRs are located in similar positions in SNAP proteins from phylogenetically distant species, we aligned the four TPR motifs (TPR1, TPR2, TPR3, and TPR4) of SNAPs from various species and obtained the corresponding phylogenetic tree. The result indicates that TPRs in equivalent positions from different species have higher similarity than TPRs within a SNAP protein (Fig. 3). For example, TPR1 from soybean GmSNAP18 is more closely related to TPR1 from the oldest plant lineage (algae *C. reinhardtii)* than to any other TPRs (TPR2 and/or TPR3 and/or TPR4) within GmSNAP18. This indicates that an original SNAP protein from the most ancestral plant lineage expanded among different species by duplication, and the same TPR motifs have been relatively well conserved throughout evolution, most likely due to functional constraints. These data suggests that SNAPs derived from a common ancestor and unique gene still present in chlorophytic algae.

### Soybean *GmSNAP* genes display overlapping and divergent functions in resistance to SCN

To investigate the specific evolutionary path(s) of different *GmSNAP* family members in soybean, we studied their expression patterns and evaluated their specific roles in response to SCN. We first compiled expression data for the *GmSNAP* genes using the public RNAseq data available at Soybase. As shown in Supplementary Figure S2, *GmSNAP* gene members presented three gene expression patterns. *GmSNAP18* and *GmSNAP11* were both ubiquitously highly expressed throughout most tissues, *GmSNAP14* had lower expression in all tissues with no expression in the leaves, and *GmSNAP02* expression only appeared in the flowers and seeds. The RNAseq analyses obtained from soybase point to a possible neofunctionalization or subfunctionalization event in the *GmSNAP* gene family in soybeans.

**Figure 4:**
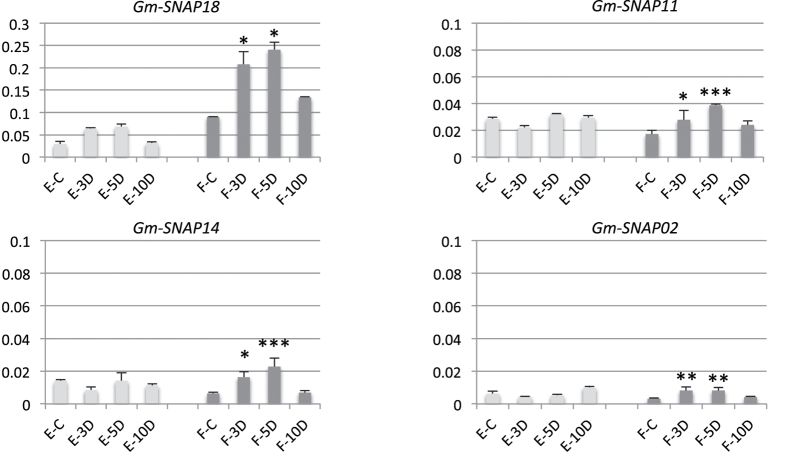
qRT-PCR of *GmSNAP* gene family in soybean in Forrest and Essex wild types. Quantitative RT-PCR analysis of four *GmSNAP* gene family members in chromosomes 02, 11, 14 and 18. Expressions were normalized using Ubiquitin as reference. (E) Essex, (F) Forrest, (C) without SCN infection, and (D) SCN infection at 3, 5 and 10 days post inoculation. The gene-specific primers designed to amplify cDNA fragments are detailed in Supplementary Table S5. *Asterisks indicate significant differences between samples as determined by t-test (***P < 0.0001, **P < 0.001, *P < 0.01).

Next, we analysed the expression using qRT-PCR of the *GmSNAP* gene members in the SCN susceptible line Essex and the SCN resistant line Forrest (Peking-type) following SCN infection. Surprisingly, the expression analysis shows that the transcripts of the four identified *GmSNAP* members in Forrest were significantly upregulated under SCN infection. Specifically, *GmSNAP18* transcripts were the most upregulated, followed by *GmSNAP11*. Furthermore, *GmSNAP14* and *GmSNAP02* presented much lower expression levels. All *GmSNAP* gene expression levels in Forrest reached the highest at five days post infection (dpi). Transcript levels were upregulated by 1.6, 1.23, 2.43, and 1.5 times for *GmSNAP18, GmSNAP11, GmSNAP14*, and *GmSNAP02*, respectively. On the contrary, a slight but not significant induction of all *GmSNAP* members was observed in the susceptible line Essex throughout the time series. Furthermore, expression levels were about 3 times more upregulated in Forrest than in Essex at 3, 5, and 10 dpi. This expression profile was maintained regardless of nematode presence (Fig. 4).

### Both *GmSNAP18* and *GmSNAP11* contribute to SCN resistance but not *GmSNAP14* and *GmSNAP02*

**Figure 5:**
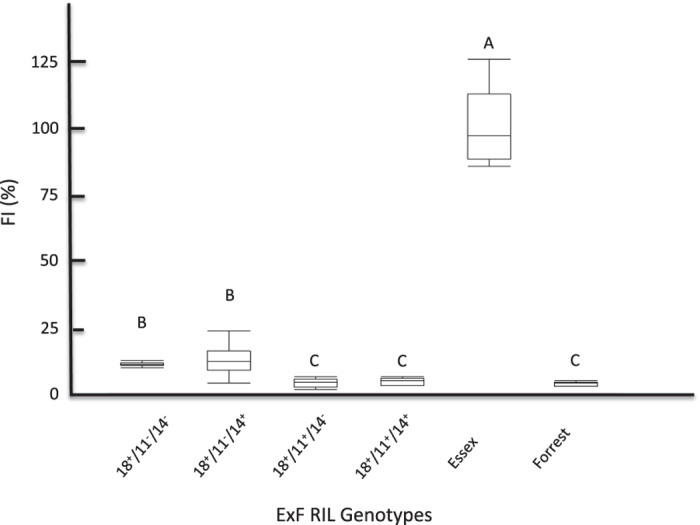
Female index presented by the four different ExF genotypes, Essex and Forrest wild types. Two lines from each genotype were analyzed. Five replicates were included for each line: n = 10 per genotype.

*α-SNAP*, corresponding to Peking-type *GmSNAP18*, has been reported to play a major role in resistance to SCN in PI88788-type soybeans (Cook *et al*.^20^). Expression analysis showed that the rest of the *GmSNAP* gene family in Forrest also responds to SCN infection. In order to test whether *GmSNAP11, GmSNAP14,* and *GmSNAP02* have the same function in SCN resistance as the identified *GmSNAP18*, and determine whether the contribution of the *GmSNAP* gene family members to SCN resistance is redundant, partially redundant, or additive, we quantified their corresponding female index (FI) using a recombinant inbred line (RIL) ExF RIL population under SCN infection^33^. In this study, the F_5_ derived RILs from the ExF population were genotyped for the following four genes: *GmSNAP18, GmSNAP11, GmSNAP14,* and *GmSNAP02*. Because *GmSNAP02* did not present any polymorphism between Forrest and Essex, the lines were classified into four different genotypes according to their allelic combinations (Supplementary Table S4). Forward screening showed a range of FI among the different ExF genotypes tested. The genotypes containing *SNAP18*^+^*/SNAP11*^+^ alleles were deemed as resistant to SCN regardless of the GmSNAP14 allele, and presented the lowest female index of 4.01% (*SNAP18*^+^*/SNAP11*^+^/*SNAP14*^−^) and 4.48% (*SNAP18*^+^*/SNAP11*^+^/*SNAP14*^+^) among the four ExF lines. A similar result was obtained in the Forrest wild type (FI = 3.65%) (Fig. 5). Surprisingly, *GmSNAP18*^+^ alone was not able to confer a complete resistance to SCN, presenting a moderate susceptibility. Thus, the female index FI = 11.05% of *SNAP18*^+^*/**SNAP11***^−^*/SNAP14*^−^ and FI = 12.59% of *SNAP18*^+^*/**SNAP11***^−^*/SNAP14*^+^ were significantly higher than the female index in the genotypes *SNAP18*^+^*/**SNAP11***^+^/*SNAP14*^−^ and *SNAP18*^+^*/**SNAP11***^+^/*SNAP14*^+^ (Fig. 5). These data point to an additive effect presented by *GmSNAP11*, but not by *GmSNAP14*. Therefore, the *GmSNAP11* at linkage group B1 is considered a novel minor contributor to SCN resistance in Peking-type alleles. However, no significant differences in FI were observed when *GmSNAP14* was present as the Forrest allele compared to the Essex allele.

## Discussion

The genome distribution of *GmSNAP* genes from syntenic analysis indicates the existence of segmental duplications and tandem rearrangement events, which occurred following an allotetraploidy event^5^,^6^,^8^,^34^. This is further supported by the evolutionary conservation of the internal modular domains among soybean GmSNAPs and phylogenetically separated SNAP proteins, and enhanced by the identification of a single *SNAP* gene in an early land plant species, i.e. *C. reinhardtii (CrSNAP*). The presence of a single *SNAP* gene in *C. reinhardtii* suggests that an ancestral aquatic SNAP protein in chlorophytic algae may have given rise to all the divergent SNAP proteins found in land plants.

Consequently, we suggest that successive duplications of a unique *SNAP* gene derived from an ancestral chlorophytic algae led to the subfunctionalization and neofunctionalization of the *SNAP* gene family in the soybean genome, contributing to the fine-tuning of soybean responses against biotic stresses, e.g. SCN resistance. Thus, the substantial changes in *SNAP* gene expression, as well as functional changes of the *SNAP* gene duplicates over time are likely due to both gene duplication and selection pressure imposed by stressful conditions. This may contribute to the physiological complexity of the soybean SNAP response against multiple stresses. These results support the hypothesis that gene duplication is an important evolutionary mechanism in the generation of novel functions and phenotypes, contributing to the adaptation of land plants to stressful environments^35^,^36^.

Furthermore, large conserved anchor numbers from the syntenic analysis revealed that *GmSNAP18* may be the most similar to the ancestral SNAP (which also gave rise to *GmSNAP14* through a duplication event that occurred (59 mya), followed by the divergence of *GmSNAP11* from *GmSNAP18*, and *GmSNAP02* from *GmSNAP14* in the most recent duplication event (13 mya). Due to the four *GmSNAP* family members: *GmSNAP02, GmSNAP11, GmSNAP14*, and *GmSNAP18* localized on syntenic genomic regions, our data suggests that the *GmSNAP* gene family evolved from a common ancestor. However, syntenic analysis did not show any duplicated block or segment between *GmSNAP18* and *GmSNAP09*. GmSNAP09 shared the lowest identity with GmSNAP18 (68%) and the highest polymorphisms on the four TPR domains compared to the rest of the four GmSNAP members. GmSNAP09 also did not conserve most of the essential carboxylate clamp residues that maintain the activity and functionality of the TPR domain. In addition, phylogenetic analysis showed that GmSNAP09 did not cluster with the other four GmSNAPs, or with the rest of the eudicot SNAPs. This suggests that GmSNAP09 may have become pseudogenized.

TPR proteins consist of three to sixteen tandem repeats of TPRs that can be grouped or dispersed throughout the protein^23^. Because most TPR proteins contain three repeats, it is likely that three is the minimum number required to form a functional domain. Our structural analysis has shown that GmSNAP proteins in soybeans are characterized by the presence of four tetratricopeptide repeats (TPR) in conserved positions along the protein (Fig. 1). This structure containing four TPR motifs supports the functionality of the TPR domain in the GmSNAPs.

Sequence analysis of many TPR proteins indicates that TPRs are defined by a pattern of small and large hydrophobic amino acids rather than a pattern of conserved amino acid residues. Although no invariant positions are found in TPRs, some amino acids tend to be conserved^9^. Three-dimensional structures have shown that a TPR motif contains two antiparallel α-helices (helix A and B), such that tandem arrays of TPR motifs generate a right-handed helical structure with an amphipathic channel that might accommodate the complementary region of a target protein^11^. Within a TPR motif, eight amino acids at positions 4 (W/L/F), 7 (L/I/M), 8 (G/A/S), 11 (Y/L/F), 20 (A/S/E), 24 (F/Y/L), 27 (A/S/L), and 32 (P/K/E) have a higher frequency of conservation and are important in maintaining the α-helical structures within a TPR motif 32. Clustering of several α-helices within a tandem array of TPR motifs generates an amphipathic channel with a large surface area, allowing the TPR domain to recognize its target protein^11^. Within these highly conserved amino acids, our *in silico* analysis shows that GmSNAP18 and GmSNAP11 conserved most of the carboxylate clamp residues (Fig. 1, Supplementary Table S3). However, GmSNAP14 and GmSNAP02 conserved less carboxylate clamp residues. This structural analysis supports the functionality of the TPR domain at GmSNAP18 and GmSNAP11 in order to recognize its target proteins and trigger the SCN resistance^32^. Interestingly, GmSNAP09 did not conserve these residues in most TPR domains. In addition, GmSNAP09 presented the highest number of polymorphisms compared to GmSNAP18 and GmSNAP11 in TPR1 (35%), TPR2 (44%), TPR3 (41%), and TPR4 (26%) (Supplementary Table S3). These data further support the evolution of GmSNAP09 obtained from the syntenic and phylogenetic analysis, and indicate that this member may have become pseudogenized.

Forward screening showed that lines with Forrest alleles (+) at both *GmSNAP18* and *GmSNAP11* were highly resistant to SCN. However, the presence of *GmSNAP18*^+^ alone was not able to confer complete resistance to SCN when the Essex allele (−) *GmSNAP11*^−^ was present. In fact, the female index of 11.05% and 12.59% obtained was significantly higher in the genotypes with *SNAP18*^+^/*SNAP11*^−^*/SNAP14*^+^ and *SNAP18*^+^/*SNAP11*^−^*/SNAP14*^−^, respectively, presenting a moderate susceptibility. Interestingly, lines containing the *GmSNAP11*^+^ allele were significantly more resistant than the ones that possessed the *GmSNAP11*^−^ allele (Fig. 5). The presence of the *GmSNAP11*^+^ Forrest allele decreases the FI in the genotypes (*SNAP18*^+^/SNAP14^+^), and (*SNAP18*^+^/*SNAP14*^−^) from 11.05% to 4.03%; and from 12.59% to 4.48%%, respectively.

The correlation between *GmSNAP11*^+^ and SCN resistance, indicate a direct link between the presence of the Forrest allele at *GmSNAP11*^+^ and increased resistance to SCN in the tested allelic combinations (Supplementary Table S4). However, this contribution is marginal and minor because of the large effect of *GmSNAP18*^+^. This finding suggests that *GmSNAP11*^+^ contributes a minor resistance to SCN, but cannot substitute the major contribution of *GmSNAP18*^+^. These data are in accordance with the results reported recently that the over-expression of an *α-SNAP* gene suppresses plant parasitic nematode infection in soybeans^19^. Using the same primers these authors used to overexpress this α-SNAP, we cloned this gene from Forrest and found that the predicted protein does not correspond to the previously reported GmSNAP18 (289 aa), but that it corresponds to a truncated GmSNAP11, which is present in Forrest Peking-type under two different types: GmSNAP11-T1 and GmSNAP11-T2, encoding a 239 aa and a 244 aa protein, respectively (Fig. 1). This difference in structure and accumulation of mutations that occurred during evolution between the two SNAP members, GmSNAP18 and GmSNAP11, explain their specific contributions to SCN resistance reported previously. Because GmSNAP11 conserves the carboxylate clamp residues and the nonsense mutations occur after the four TPR motifs, but before the two reported polymorphisms between resistant and susceptible lines at the C-terminus, both GmSNAP11 truncated proteins may conserve protein-protein interaction capabilities, but may lose the downstream activity and affect its function (Fig. 5). Thus, the *GmSNAP11* expression profiles, structural, and SCN phenotypic analysis point to the discovery of a minor contribution of *GmSNAP11* for resistance to SCN.

After duplication, the predominant fate of duplicated genes is pseudogenization^37^. Still, a significant fraction of duplicated genes in plants are preserved and follow different evolutionary paths including retention, neofunctionalization, and subfunctionalization. The phylogenetic analysis within the soybean SNAP family indicates that GmSNAP18 and GmSNAP11 proteins cluster together and form a separate clade from GmSNAP14 and GmSNAP02 (Fig. 2). Because GmSNAP11 and GmSNAP18 began diverging recently, they are closely related with a 92.4% amino acid sequence similarity. However, nonsense mutations in GmSNAP11 at positions E244* and A240* result in a truncated protein which may affect and reduce the function of GmSNAP11-T1 and GmSNAP11-T2 in Peking-type Forrest, without entirely supressing its function. Previous studies in the plant model *Arabidopsis thaliana* have shown that a TPR protein family named Tetratricopeptide repeat thioredoxin-like (TTL), formed by four members. TTL1, TTL3, and TTL4, but not TTL2, presented an additive effect in response to osmotic stress tolerance and are essential for root growth and integrity, while all present the same amino acid sequence length with high identity^12^. This is an example of a duplicated gene resulting in a truncated protein (50 aa less) that presents an additive effect in resistance to a pathogen.

Furthermore, the reported low differences in *GmSNAP14* and *GmSNAP02* expression patterns and function suggest that its subfunctionalization is ongoing. It has been reported that in addition to *GmSNAP18, GmSNAP11* and *GmSNAP14* were associated with QTL for resistance to SCN^38^,^39^. *GmSNAP02* was reported to be associated with QTL for resistance to *Phytophtora*^40^. Moreover, *GmSNAP02* RNAseq data (soybase RNAseq expression) show that its expression was confined only to flowers and seeds, and qRT-PCR data showed that *GmSNAP02* had a very low expression in the roots, which suggests that it may have been subfunctionalized. Similar results were reported in the TPR protein TTL2, which was demonstrated to have neofunctionalized to be involved in male sporogenesis^12^. TTL2 is essential for gametophytic viability but not in root growth and integrity under osmotic stress, as is the rest of the TTL gene family^12^. Furthermore, the expression data shows that the four closely related *GmSNAP*s may have acquired an accumulation of mutations that lead to a difference in their regulatory or protein sequences, ultimately leading to a neofunctionalization or subfunctionalization. However, these genes are generally responding to the same controlling elements, which could directly or indirectly trigger their expression. The presence of the cumulated mutations within the TPR domains of each reported *GmSNAP* member and the differences within their C-termini and the rest of the protein sequence are most likely to dictate potential interaction and localization preferences (Fig. 1).

Although the roles of *GmSNAP14* and *GmSNAP02* members needs to be elucidated, this study shows evidence of the neofunctionalization and subfunctionalization of the *GmSNAP* gene family in Peking-type soybeans. Considering the function of soybean *GmSNAP18* in SCN resistance^20^, and the newly discovered function of *GmSNAP11* as a minor contributor to SCN resistance, the duplication and retention of *SNAP* genes in plants suggests that *SNAP* genes may be a source of diversity which is important for proper responses to stressful environments.

## Material and Methods

### SCN-infection phenotyping

SCN-infection phenotyping was performed on the M3 lines as described by ref. 41. Seedlings were inoculated with infective eggs from the PA3 population (HG type 0). Briefly, cysts were extracted from Essex infested roots and soil by flotation in water and collected on a 250-μm sieve. Harvested cysts were gently crushed using a drill press and the eggs were collected on a 25-μm sieve^42^. The eggs were further diluted to 1,000 eggs/ml of water. Individual seedlings were inoculated with 1 ml of the egg suspension. Plants were maintained in the growth chamber at 27 °C. Cyst counts were performed at 30 days post inoculation.

### Phylogenetic Analysis and Genomic Structure

Multiple sequence alignments were performed using the MEGA4 software package and the Clustal W algorithm. An unrooted phylogenetic tree was calculated with the neighbor-joining method^43^, and tree topology robustness was tested by bootstrap analysis of 1,000 replicates. Alignment analysis of SNAPs in soybean (*Glycine max* (L.) Merr.), Arabidopsis *Arabidopsis thaliana* (dicotyledons model), rice *Oryza sativa* (monocotyledons model), *Physcomitrella patens* (moss model), *Selaginella moellendorffii* (lycophyte model), *Chlamydomonas reinhardtii* (algae model), in addition to other monocots (*H. vulgare, S. italica, Z. mays, S. bicolor*), and eudicots (*C. sativus, T. cacao, P. trichocarpa, R. communis, P. persica, C. clementine, C. sinensis, E. grandis, A. thaliana, V. vinifera, S. lycopersicum*), were obtained using MegAlign 4 software. SNAP and TPR domain *in silico* analysis was performed using the MegAlign (DNASTAR Lasergene 8) software package and the Clustal W algorithm. All parameter values correspond to default definitions.

### Genotyping of ExF RILs population

The ExF population containing 100 RILs used in this study was developed at Southern Illinois University Carbondale^44^. The Eco TILLING marker *GmSNAP18, GmSNAP11, GmSNAP14*, and *GmSNAP02* whose primers were listed in Supplementary Table S5, were developed and used to identify the genotype of each ExF RIL at the genes *GmSNAP18* (Glyma.18G022500), *GmSNAP11* (Glyma.11G234500), *GmSNAP02* (Glyma.02G260400), and *GmSNAP14* (Glyma.14G054900) by EcoTILLING. The EcoTILLING was conducted as described by ref. 41.

### SNAP Analysis

The mining of SNAP genes was performed by searching sequences homologous to the GmSNAP18 proteins using the Phytozome database (www.phytozome.net). SNAP sequences obtained from different species were also analyzed using the NCBI Conserved Domain Database for TPR domains (http://www.ncbi.nlm.nih.gov/Structure/cdd/cdd.shtml). Generation of the unrooted phylogenetic tree was performed by the alignment of the full-length amino acid sequences using ClustalW and the DNASTAR Lasergene 8 software.

### qRT-PCR of *GmSNAP* gene family

Soybean seedlings from the susceptible line Essex and the resistant lines Forrest (wild types) were grown in autoclaved sandy soil in the growth chamber for one week and then infected with eggs from the PA3 population. Total RNA was isolated from root samples after three, five, and ten days following SCN infection using a Qiagen RNeasy Plant Mini Kit (cat# 74904). Total RNA was DNase treated and purified using Turbo DNA-free Kit (QAmbion/life technologies AM1907). RNA was quantified using Nanodrop 1000 (V3.7). Then, a total of 400 nanograms of treated RNA was used to generate cDNA, using the cDNA synthesis kit (Thermoscript, life technologies, 11146-025) with random hexamers and 1/10th of a 20 microliter RT reaction was used in gene-specific quantitative PCR with the Power SYBR^®^ Green PCR Master Mix kit (Applied Biosystems™ #4368706). A list of primers used in this work is found in Supplementary Table S5. For each genotype/primer pair, RNA from three individual biological replicates was used for quantitation and then normalized using the delta C_q_ method with Ubiquitin used as a reference gene (ΔCq = C_q(TAR)_ − C_q(REF)_). Each gene’s expression was exponentially transformed to the expression level using the formula (ΔCq Expression = 2^−ΔCq^). A –RT reaction was also performed in all the samples.

### Bioinformatics Analysis

Bioinformatics analysis was performed using the following databases: The Soybean database (www.soybase.org), NCBI (www.ncbi.nlm.nih.gov), Plant Genome Duplication Database (http://chibba.agtec.uga.edu/duplication/), phytosome (www.phytozome.net) and the European Bioinformatics Institute (www.ebi.ac.uk/Tools/clustalw2/index.html).

### Statistical analysis

All presented results were performed with the analysis of variance by a T-student test means comparison, using JMP Pro V12 software.

### Ethics and consent to participate

This study did not involve humans, human data or animals; no ethics approval or consent is required to publish the results.

### Availability of data and materials

The developed cross between the resistant Forrest (+) and the susceptible Essex (−) using both recombinant inbred lines (RILs) and near-isogenic lines (NILs) seeds, are property of Southern Illinois University (SIU). Access to the germplasm is subjected to Transfer Material Agreement Form.

## Additional Information

**How to cite this article:** Lakhssassi, N. *et al*. Characterization of the Soluble NSF Attachment Protein gene family identifies two members involved in additive resistance to a plant pathogen. *Sci. Rep.* **7**, 45226; doi: 10.1038/srep45226 (2017).

**Publisher's note:** Springer Nature remains neutral with regard to jurisdictional claims in published maps and institutional affiliations.

## Supplementary Material

Supplementary Files

## Figures and Tables

**Table 1:** Gene divergence of duplicated regions around *GmSNAP* gene members in soybean between chr18 (1.54–1.74 Kb), chr11 (32.87–33.07 Kb), chr14 (4.28–4.48 Kb), and chr02 (44.6–44.8 Kb).

Locus 1	Annotation 1	Block order	Locus 2	Ka	Ks	Ka/Ks	Block order	Locus 3	Ka	Ks	Ka/Ks	Block order	Locus 4	Ka	Ks	Ka/Ks
Glyma.18G021200	EXPRESSED PROTEIN	200	Glyma.11G235700	0.03	0.2	0.15										
Glyma.18G021300	Weak chloroplast movement under blue light (WEMBL)	201	Glyma.11G235600	0.04	0.16	0.25	1	Glyma.02G259100	0.22	0.71	0.31					
Glyma.18G021400	HALOACID DEHALOGENASE-LIKE HYDROLASE	202	Glyma.11G235500	0.08	0.21	0.38										
Glyma.18G021500	Uroporphyrinogen decarboxylase	203	Glyma.11G235400	0.06	0.1	0.60										
Glyma.18G021600	CBL-INTERACTING SERINE/THREONINE-PROTEIN KINASE 2	204	Glyma.11G235300	0.02	0.1	0.20										
Glyma.18G021700	PROTEIN NRT1/PTR FAMILY 5.1	205	Glyma.11G235200	0.01	0.12	0.08	2	Glyma.02G259400	0.11	0.6	0.18					
Glyma.18G022000	Hypoxia induced protein conserved region (HIG_1_N)	206	Glyma.11G235000	0.09	0.15	0.60	3	Glyma.02G259700	0.11	0.79	0.14					
Glyma.18G022100	BTB/POZ domain	207	Glyma.11G234900	0.05	0.06	0.83										
Glyma.18G022200	Unknown Function	208	Glyma.11G234800	0.04	0.13	0.31	4	Glyma.02G259800	0.12	0.37	0.32	4	Glyma.14G054100	0.15	0.67	0.22
Glyma.18G022300	BETA CATENIN-RELATED ARMADILLO REPEAT-CONTAINING	209	Glyma.11G234700	0.03	0.14	0.21										
Glyma.18G022400	AMINO ACID TRANSPORTER	210	Glyma.11G234600	0.06	0.14	0.43	5	Glyma.02G260100	0.16	0.63	0.25					
**Glyma.18G022500**	**SOLUBLE NSF ATTACHMENT PROTEIN (GmSNAP18)**	**211**	**Glyma.11G234500**	**0.01**	**0.13**	**0.08**	**6**	**Glyma.02G260400**	**0.07**	**0.56**	**0.13**	**5**	**Glyma.14G054900**	**0.06**	**0.53**	**0.11**
Glyma.18G022600	(Z)-GAMMA-BISABOLENE SYNTHASE 1-RELATED	212	Glyma.11G234400	0.02	0.13	0.15	7	Glyma.02G260500	0.14	0.47	0.30					
Glyma.18G022700	SnoaL-like domain	213	Glyma.11G234300	0.02	0.14	0.14										
Glyma.18G022800	Unknown Function	214	Glyma.11G234200	0.04	0.09	0.44										
Glyma.18G022900	HEAVY METAL TRANSPORT/DETOXIFICATION SUPERFAMILY	215	Glyma.11G234100	0.02	0.15	0.13	8	Glyma.02G260700	0.13	0.65	0.20	6	Glyma.14G055100	0.1	0.91	0.11
Glyma.18G023000	Sodium/hydrogen exchanger family (Na_H_Exchanger)	216	Glyma.11G234000	0.03	0.03	1.00										
Glyma.18G023100	Arogenate dehydrogenase (NADP(+))/TyrAAT2	217	Glyma.11G233900	0.01	0.12	0.08	9	Glyma.02G260900	0.2	0.78	0.26	7	Glyma.14G055300	0.2	1.14	0.18
Glyma.18G023200	Haem-binding uptake, Tiki superfamily, ChaN (Cofac_haem_bdg)	218	Glyma.11G233800	0.02	0.09	0.22										
Glyma.18G023300	CARBOXYLASE:PYRUVATE/ACETYL-COA/PROPIONYL-COA CARBOXYLASE	219	Glyma.11G233700	0.01	0.07	0.14										
Glyma.18G023400	Unknown Function	220	Glyma.11G233600	0.01	0.07	0.14	10	Glyma.02G261000	0.24	0.8	0.30	8	Glyma.14G055400	0.23	0.83	0.28
Glyma.18G023500	LEUCINE-RICH REPEAT RECEPTOR-LIKE PROTEIN KINASE IMK3-RELATED	221	Glyma.11G233500	0.04	0.13	0.31	11	Glyma.02G261400	0.12	0.57	0.21	9	Glyma.14G055900	0.12	0.56	0.21
Glyma.18G023600	LACCASE-12-RELATED	222	Glyma.11G233400	0.07	0.18	0.39	12	Glyma.02G261600	0.07	0.74	0.09	10	Glyma.14G056100	0.07	0.73	0.10

Analysis represent +/− 100 kb duplicated region centered in the *GmSNAP* genes.
