# Supplementary material for: Characterization of the Soluble NSF Attachment Protein gene family identifies two members involved in additive resistance to a plant pathogen
Source: Sci Rep. 2017 Mar 24;7:45226. doi: 10.1038/srep45226 (PMC5364553; doi:10.1038/srep45226)
Supplement: Supplementary Files [file srep45226-s1.pdf]

Characterization of the Soluble NSF Attachment Protein gene family identifies two members involved in additive resistance to a plant pathogen

Naoufal Lakhssassi[1], Shiming Liu[1†], Sadia Bekal[1†], Zhou Zhou[1], Vincent Colantonio[1], Kris Lambert[2], Abdelali Barakat[3], and Khalid Meksem[1*]

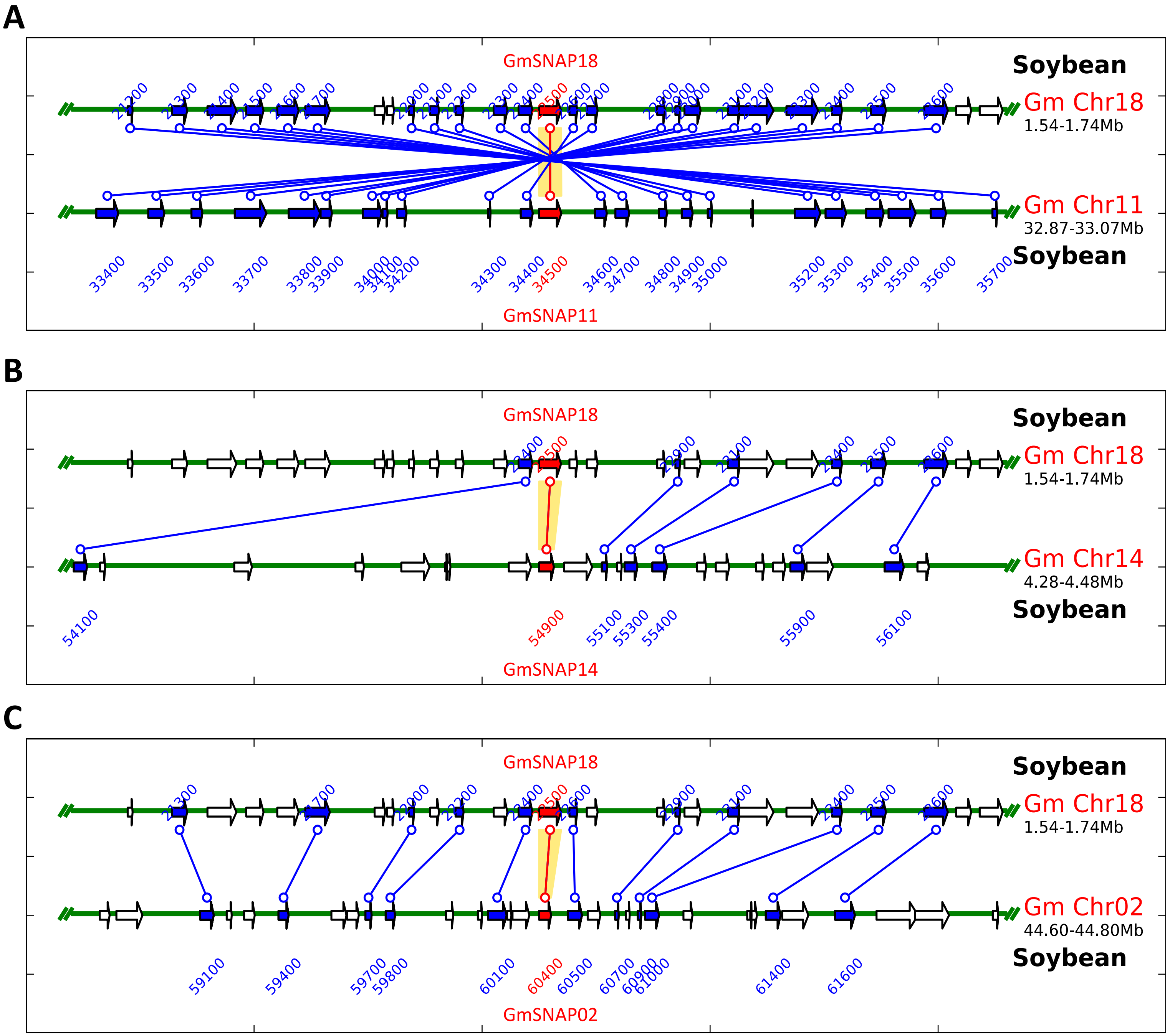

Supplementary Figure S1. Schematic representation of *GmSNAP* containing duplicated segments identified in the soybean genome. Soybean *SNAP* intragenome syntenic relationships were calculated using the Plant Genome Duplication Database. (**A**) *GmSNAP* in chr18 and chr11 belongs to a very large inverted duplicated segment containing 22 additional conserved duplicated genes or anchors. (**B**) *GmSNAP* in chr18 and chr14 belongs to another duplicated segment with less conservation; just 6 additional duplicated genes or anchors. (**C**) *GmSNAP* in chr18 and chr02 belongs to a third duplicated segment containing 11 additional duplicated genes or anchors. Graphs represent +/- 100 kb duplicated region centred in the *GmSNAP* genes.

Supplementary Table S1. Number of *SNAP* genes identified in select algae, moss, lycophyte, basal angiosperms, monocotyledonous, and dicotyledonous species available in Phytozome (www.phytozome.net).

Species	Number of SNAPs	Accession Number	Length	NCBI Description (Functional Domains)
**Red Algae**				
Hemiselmis andersenii	0	-	-	-
**Green Algae**				
Chlamydomonas reinhardtii	1	XP_001700026.1	306aa	Alpha-SNAP (Four TPRs)
Ostreococcus lucimarinus	1	XP_001422423.1	303aa	Predicted protein (Four TPRs)
**Moss**				
Physcomitrella patens	4	XP_001775764.1	293aa	Predicted protein (Four TPRs)
		XP_001752311.1	287aa	Predicted protein (Four TPRs)
		XP_001754124.1	290aa	Predicted protein (Four TPRs)
		XP_001772250.1	290aa	Predicted protein (Four TPRs)
**Lycophyte**				
Selaginella moellendorffii	4	XP_002980834.1	288aa	hypothetical protein (Four TPRs)
		XP_002989373.1	288aa	hypothetical protein (Four TPRs)
		XP_002970299.1	289aa	hypothetical protein (Four TPRs)
		XP_002978461.1	289aa	hypothetical protein (Four TPRs)
**Basal Angiosperm**				
Amborella trichopoda	1	XP_006843687.1	289aa	PREDICTED: alpha-soluble NSF attachment protein
**Monocotyledons**				
Oryza sativa Japónica	1	LOC_Os08g18110	289aa	Alpha-soluble NSF attachment protein (Four TPRs)
Zea mays	2	GRMZM2G098496_T01	289aa	Alpha-soluble NSF attachment protein (Four TPRs)
		GRMZM2G011559_T01	289aa	Alpha-soluble NSF attachment protein (Four TPRs)
Setaria italica	3	Seita.6G107400.1	289aa	Alpha-soluble NSF attachment protein (Four TPRs)
		Seita.9G179000.1	289aa	Alpha-soluble NSF attachment protein (Four TPRs)
		Seita.1G261500.1	214aa	Alpha-soluble NSF attachment protein (Four TPRs)
Sorghum bicolor	1	Sobic.007G114200.1	289aa	Alpha-soluble NSF attachment protein (Four TPRs)
**Dicotyledons**				
Arabidopsis thaliana	1	AT3G56190	289aa	Alpha-soluble NSF attachment protein (Four TPRs)
Glycine max	5	Glyma.18g022500	289aa	Alpha-soluble NSF attachment protein (Four TPRs)
		Glyma.11g234500	289aa	Alpha-soluble NSF attachment protein (Four TPRs)
		Glyma.14G054900	291aa	Alpha-soluble NSF attachment protein (Four TPRs)
		Glyma.02G260400	291aa	Alpha-soluble NSF attachment protein (Four TPRs)
		Glyma.09G279400	292aa	Alpha-soluble NSF attachment protein (Four TPRs)
Vitis vinifera	2	GSVIVT01032725001	289aa	Alpha-soluble NSF attachment protein (Four TPRs)
		GSVIVT01030239001	289aa	Alpha-soluble NSF attachment protein (Four TPRs)
Prunus persica	1	Prupe.2G259200.1	289aa	Alpha-soluble NSF attachment protein (Four TPRs)
Cucumis sativus	1	Cucsa.326170.1	289aa	Alpha-soluble NSF attachment protein (Four TPRs)
Ricinus communis	1	29729.m002318	289aa	Alpha-soluble NSF attachment protein (Four TPRs)
Solanum tuberosum	3	PGSC0003DMT400089090	307aa	Alpha-soluble NSF attachment protein (Four TPRs)
		PGSC0003DMT400046100	289aa	Alpha-soluble NSF attachment protein (Four TPRs)
		PGSC0003DMT400030420	289aa	Alpha-soluble NSF attachment protein (Four TPRs)
Solanum lycopersicum	2	Solyc06g050770.2	289aa	Alpha-soluble NSF attachment protein (Four TPRs)
		Solyc05g052310.2	243aa	Alpha-soluble NSF attachment protein (Four TPRs)
Citrus sinensis	1	orange1.1g022992m	289aa	Alpha-soluble NSF attachment protein (Four TPRs)
Theobroma cacao	1	Thecc1EG017057t1	289aa	Alpha-soluble NSF attachment protein (Four TPRs)
Citrus clementina	1	Ciclev10032291m	289aa	Alpha-soluble NSF attachment protein (Four TPRs)
Eucalyptus grandis	2	Eucgr.G03107.1	289aa	Alpha-soluble NSF attachment protein (Four TPRs)
		Eucgr.D01340.1	218aa	Alpha-soluble NSF attachment protein (Four TPRs)
Populus trichocarpa	3	Potri.016G129900.1	288aa	Alpha-soluble NSF attachment protein (Four TPRs)
		Potri.010G181900.1	288aa	Alpha-soluble NSF attachment protein (Four TPRs)
		Potri.008G075400.1	288aa	Alpha-soluble NSF attachment protein (Four TPRs)

Supplementary Table S2. *GmSNAP*s intragenome syntenic relationship calculations showing independent duplicate blocks containing the genomic pairs of *GmSNAP* family members with their corresponding number of conserved genes or anchors. Soybean genome duplicated chromosomal segments containing *GmSNAP*s were calculated using the Plant Genome Duplication.

Gene A	Gene B	Score	E-Value	Block	Conserved Anchors
SNAP18	***SNAP11***	15353	0	huge	**386**
SNAP18	*SNAP14*	2818	0	huge	72
SNAP18	*SNAP02*	901	0	large	23
SNAP11	*SNAP14*	2618	3.00E-124	huge	67
SNAP11	*SNAP02*	821	0	large	21
SNAP14	***SNAP02***	1469	1.00E-49	large	**37**

Supplementary Table S3. Polymorphisms presented by the five identified predicted GmSNAP protein family members in the four TPR domains. Percentage of polymorphism was calculated using GmSNAP18 as reference.

GmSNAPs	TPR1														Poly %
GmSNAP02	-	-	A7S	-	C10S	F11Y	-	A22S	-	-	L25I	-	-	-	14.70
GmSNAP14	-	-	A7S	-	C10S	F11Y	-	A22S	-	-	L25I	-	S29N	H31N	20.58
GmSNAP11	-	-	-	-	-	-	-	-	-	-	-	-	-	-	-
GmSNAP18	-	-	-	-	-	-	-	-	-	-	-	-	-	-	-
GmSNAP09	D2E	D5H	A7S	N9T	C10S	-	G21A	A22S	T23L	Y24F	L25V	L27S	S29K	-	**35.29**

GmSNAPs	TPR2																Poly %
GmSNAP02	-	H4Y	H9R	S10C	-	-	-	-	-	-	-	R25Q	-	L29I	-	D32E	17.64
GmSNAP14	-	H4Y	-	S10C	-	-	-	-	-	-	-	R25N	-	L29I	-	D32E	14.70
GmSNAP11	-	-	-	-	-	-	-	-	-	-	-	R25Q	-	-	-	-	2.94
GmSNAP18	-	-	-	-	-	-	-	-	-	-	-	-	-	-	-	-	-
GmSNAP09	Q2N	H4Y	-	S10C	N15S	I16T	N17S	E18G	S19A	V20I	D24N	R25K	N28T	L29I	C31T	D32E	**44.11**

GmSNAPs	TPR3																Poly %
GmSNAP02	-	-	-	-	G16S	-	-	E21S	-	L24V	V25A	-	-	-	-	-	11.76
GmSNAP14	-	-	-	-	G16S	-	-	-	-	L24V	-	-	-	-	-	F34Y	8.23
GmSNAP11	-	-	-	-	-	-	-	-	-	-	-	-	-	-	-	-	-
GmSNAP18	-	-	-	-	-	-	-	-	-	-	-	-	-	-	-	-	-
GmSNAP09	S1I	R5K	L7S	A11G	G16L	E17D	N19D	-	Q22H	L24R	V25S	K29R	S30A	D32E	F33L	-	**41.17**

GmSNAPs	TPR4											Poly %
GmSNAP02	-	-	A11S	-	K19R	-	D22E	E26D	-	N33S	-	14.70
GmSNAP14	-	-	A11S	-	K19R	-	D22E	E26D	-	N33S	-	14.70
GmSNAP11	-	-	-	-	-	-	D22E	-	A28T	-	-	5.88
GmSNAP18	-	-	-	-	-	-	-	-	-	-	-	
GmSNAP09	N1I	Q5V	A11C	E15Q	-	S20A	D22K	E26D	-	N33D	N34S	**26.47**

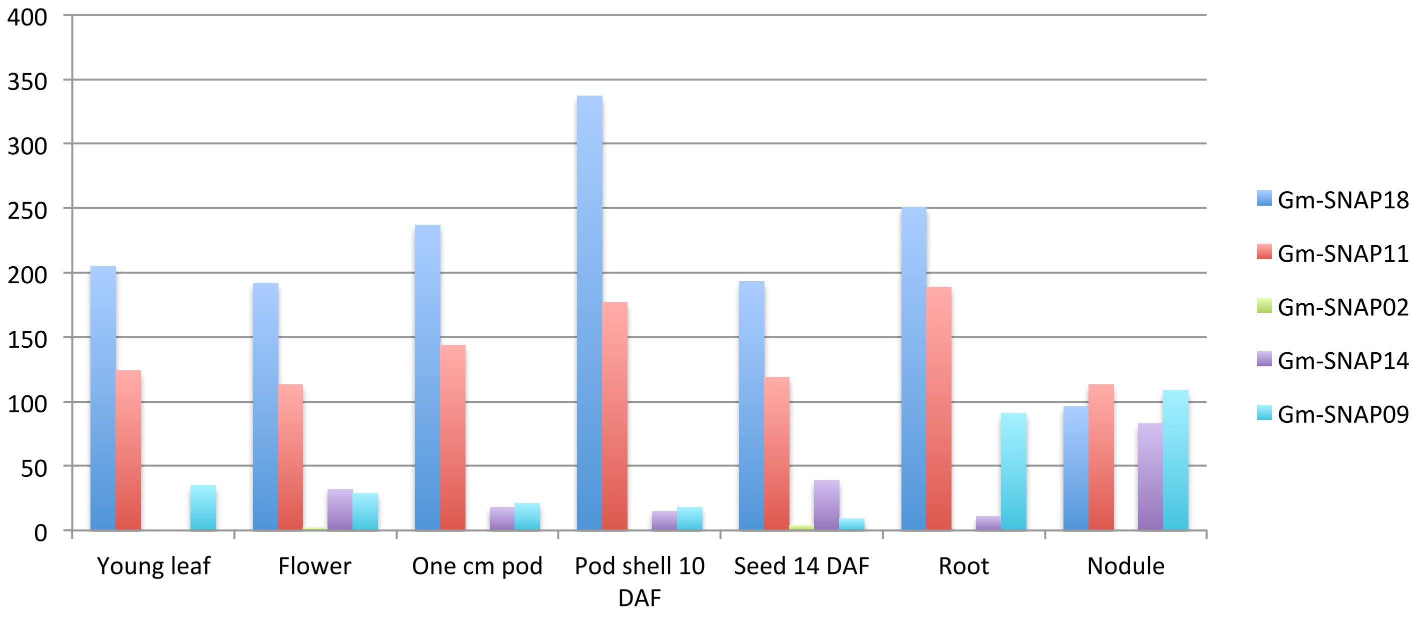

Supplementary Figure S2. Expression pattern of the five soybean *GmSNAP* gene members in planta, based on Soyseq resource available from RNAsequencing data (http://www.soybase.org/soyseq).

Supplementary Table S4. Genotypes of the F5 derived RILs from the ExF population for three genes: *GmSNAP18, GmSNAP11*, and *GmSNAP14*. *GmSNAP02* was identical between Essex and Forrest, and thus, was not considered. The lines were classified into four different genotypes according to their allelic combinations.

RILs	GmSNAP18	GmSNAP11	GmSNAP14
$18^+/11^-/14^-$	Forrest	Essex	Essex
$18^+/11^-/14^+$	Forrest	Essex	Forrest
$18^+/11^+/14^-$	Forrest	Forrest	Essex
$18^+/11^+/14^+$	Forrest	Forrest	Forrest

Supplementary Table S5. Primers used for genotyping, expression (qRT-PCR), sequencing and EcoTILLING.

Gene	OLD GENE ID V1.1	Gene model	Primers	Primer Sequences	Purpose
GmSNAP18	Glyma18g02590	Glyma.18G022500	GmSNAP18-RT-Fw	ACAAGGCTGGAGCGACATAC	qRT-PCR
			GmSNAP18-RT-Rv	AGCAATGTGCAGCATCGACA	
			GmSNAP18-Fw	CACTGTGTAAAGTTAATTTTTTTGCTTAC	EcoTILLING
			GmSNAP18-Rv	CCAATTCAATTAAAACCAAAGCAGG	
Gm-SNAP11	Glyma11g35820	Glyma.11G234500	GmSNAP11-RT-Fw	AATATCAGAAGTCGATTGAGATTTAC	qRT-PCR
			GmSNAP11-RT-Rv	ATTGGTTACAGCAATAACATCCC	
			GmSNAP11-Fw	GAATCTTGATGATACACAGCCTTG	EcoTILLING
			GmSNAP11-Rv	GCACACATGCAATGTTGATGAGCT	
GmSNAP14	Glyma14g05920	Glyma.14G054900	GmSNAP14-RT-Fw	ATATCGAGCAGGCCGTTGTT	qRT-PCR
			GmSNAP14-RT-Rv	GCACTGGTTTGCAGAAGTGT	
			GmSNAP14-Fw	GGCTATCCCTTTTGAGGAATTCT	EcoTILLING
			GmSNAP14-Rv	TCACGTGCAAGCGGTGCACATA	
GmSNAP02	Glyma02g42820	Glyma.02G260400	GmSNAP02-RT-Fw	CCAGGGCCGAGGATTTTGAG	qRT-PCR
			GmSNAP02-RT-Rv	TCGAAGAGATCAGCAGCGTC	
			GmSNAP02-Fw1	GAGAGAGCATTAAATTGAATTGATCG	EcoTILLING
			GmSNAP02-Rv1	CATATAGAGAAGGCTATTATAAAGTTTC	
			GmSNAP02-Fw2	CCATTCATTGCTGCATTTAATCTTATTC	
			GmSNAP02-Rv2	ATGACAACTCATGGAGATATGC	
Gm-Ubiqutin	Glyma20g27950	Glyma.20G141600	GmUBI20-RT-Fw	GTGTAATGTTGGATGTGTTCCC	qRT-PCR
			GmUBI20-RT-Rv	ACACAATTGAGTTCAACACAAACCG	
